# Iterative framework for image registration and partial volume correction in brain positron emission tomography

**DOI:** 10.1007/s12194-020-00591-2

**Published:** 2020-10-19

**Authors:** Keisuke Matsubara, Masanobu Ibaraki, Miho Shidahara, Toshibumi Kinoshita, for the Alzheimer’s Disease Neuroimaging Initiative

**Affiliations:** 1 Department of Radiology and Nuclear Medicine, Research Institute for Brain and Blood Vessels, Akita Cerebrospinal and Cardiovascular Center, 6-10 Senshu-Kubota-machi, Akita, 010-0874 Japan; 2 Department of Quantum Science and Energy Engineering, Graduate School of Engineering, Tohoku University, Sendai, Japan

**Keywords:** Amyloid, Image registration, Partial volume correction, PET

## Abstract

Imprecise registration between positron emission tomography (PET) and anatomical magnetic resonance (MR) images is a critical source of error in MR imaging-guided partial volume correction (MR-PVC). Here, we propose a novel framework for image registration and partial volume correction, which we term PVC-optimized registration (PoR), to address imprecise registration. The PoR framework iterates PVC and registration between uncorrected PET and smoothed PV-corrected images to obtain precise registration. We applied PoR to the [^11^C]PiB PET data of 92 participants obtained from the Alzheimer’s Disease Neuroimaging Initiative database and compared the registration results, PV-corrected standardized uptake value (SUV) and its ratio to the cerebellum (SUVR), and intra-region coefficient of variation (CoV) between PoR and conventional registration. Significant differences in registration of as much as 2.74 mm and 3.02° were observed between the two methods (effect size <  − 0.8 or > 0.8), which resulted in considerable SUVR differences throughout the brain, reaching a maximal difference of 62.3% in the sensory motor cortex. Intra-region CoV was significantly reduced using the PoR throughout the brain. These results suggest that PoR reduces error as a result of imprecise registration in PVC and is a useful method for accurately quantifying the amyloid burden in PET.

**Electronic supplementary material:**

The online version of this article (10.1007/s12194-020-00591-2) contains supplementary material, which is available to authorized users.

## Introduction

Positron emission tomography (PET) has been used to quantify biological processes in the cerebral cortex, particularly the deposition of amyloid beta plaques [1–4] and neurofibrillary tangles [5–8], which occur in neurodegenerative disorders, including Alzheimer’s disease (AD). The low spatial resolution of PET measurements, typically 5–8 mm at full-width half-maximum (FWHM), results in spill-out radioactivity concentration from the region of interest (ROI) and spill-in from marginal regions; this phenomenon is known as a “partial volume effect” [9]. Morphological changes to ROIs increase the partial volume effect. For example, thinning of the cortical gyri caused by atrophy results in a stronger spill-out from gray matter (GM) regions, thereby leading to an underestimation of the concentration of cortical radioactivity. Furthermore, particularly in amyloid PET, radioligands for amyloid PET are highly accumulated in white matter (WM) regions [1, 10], regardless of amyloid deposition. The spillover from WM can result in an overestimation of uptake, particularly in cortical GM without amyloid deposition [11]. This fact implies the need to correct the spillover from WM as well as that from GM caused by cortical atrophy for quantitative and cross-sectional studies with amyloid PET.

Several anatomical imaging-guided partial volume correction (PVC) methods employing magnetic resonance (MR) and computed tomography (CT) imaging have been proposed to correct partial volume effects [12–19]. For example, in Müller–Gärtner’s method, tissue fractions for GM, WM, and cerebrospinal fluid (CSF) estimated using MR segmentation are used to estimate spillover from WM and CSF to GM before calculating the PV-corrected radioactivity concentration for GM [16]. The geometric transfer matrix (GTM) method proposed by Rousset et al*.* involves calculation of a matrix including spillover among ROIs, followed by performing region-wise PVC [17]. Recently, an extension of the GTM method to voxel-wise PVC has been proposed [19]. Some MR-imaging-guided PVC (MR-PVC) methods have been implemented in software packages such as PMOD (https://www.pmod.com/web/) and FreeSurfer (https://surfer.nmr.mgh.harvard.edu/fswiki/PetSurfer). These methods are widely used in brain PET studies [20–24].

MR-PVC is sensitive to misregistration between PET and MR images [14]. Therefore, precise registration is required for this method. Previous systematic validations have demonstrated that registration errors result in biases in the GM radioactivity concentration estimated by Müller–Gärtner’s method [25, 26]. In GTM methods, high variance [27] and bias [28] in PV-corrected radioactivity concentration estimates caused by registration error have been reported. However, to date, no comprehensive solution has been proposed to avoid registration errors in MR-PVC.

Previous reports involving amyloid PET studies have demonstrated that MR-PVC can improve the statistical power in cross-sectional [19, 29] and longitudinal [30] analyses. However, an increase in inter-scan variability due to noise amplification and other erroneous factors in PVC and the subsequent reduction in statistical power in longitudinal analysis have previously been reported [31]. Therefore, whether PVC should be applied for accurate quantification of amyloid with PET remains an open question.

We speculated that avoiding registration error can improve the accuracy of quantification for amyloid deposition using PET and MR-PVC. In this study, we propose a novel framework termed PVC-optimized registration (PoR) to address registration errors in MR-PVC. We assumed that incomplete registration between MR and PET images results in a discrepancy between the registered PET images and map corrected for the partial volume effect (PV-corrected). To reduce the discrepancy due to incomplete registration, in the PoR framework, registration is iteratively performed between the uncorrected PET image and the map PV-corrected and then smoothed with a point spread function (PSF) for the PET images. Theoretically, PoR can converge to accurate registration between PET and MR spaces if PVC is appropriately performed, because the smoothed PV-corrected map involves anatomical information from the MR image as well as similar contrast to the original PET image. To validate the impact of the PoR framework, we applied PoR to [^11^C]PiB PET data obtained from the Alzheimer’s Disease Neuroimaging Initiative (ADNI) database and compared the results to conventional registration.

## Materials and methods

### Data

In this study, data were obtained from the ADNI database, which was launched in 2003 as a public–private partnership led by Principal Investigator Michael W. Weiner, MD. ADNI primarily aimed to investigate whether a combination of measurements from serial MRI, PET, clinical and neuropsychological assessments, and other biological markers can be used to measure the progression of mild cognitive impairment (MCI) and early AD (for up-to-date information, please see www.adni-info.org).

We analyzed data acquired from 92 participants, including 16 healthy controls, 58 patients with MCI, and 18 patients with AD. The first PiB PET and MR 3D T1-weighted scans for each participant were selected for analysis.

### Preprocessing

For PVC processing of images with raw voxel values, we downloaded PET data that were preprocessed by registration of each frame to the first frame, and the frames were averaged (5 min × 4 frames starting at 50 min after [^11^C]PiB injection; termed “Coregister, Averaged” in the ADNI database). Downloaded PET images were smoothed using a Gaussian kernel applied to the “post-processed” image (namely, “Co-reg, Avg, Std Img, and Vox Siz, Uniform resolution” in the ADNI database) in each ADNI site. The images and voxel sizes of the downloaded PET images are shown in Table S1 in the Supplementary Materials. The sizes of the Gaussian kernel are shown in Table S2 in the Supplementary Materials. The smoothed PET images had a uniform isotropic resolution of 8 mm FWHM. Therefore, a point spread function (PSF) using an isotropic Gaussian kernel of 8 mm FWHM was assumed for all the PVC in this study.

MR T1-weighted images were analyzed using FreeSurfer (https://surfer.nmr.mgh.harvard.edu) for automatic labeling of volumes of interest (VOIs) [32, 33]. The MR T1-weighted images were resampled to 256 × 256 × 256 voxels with a 1 × 1 × 1 mm^3^ voxel before the automatic labeling. Overall, 113 VOIs labeled by FreeSurfer and derived from the Desikan/Killiany atlas (aparc + aseg) [34] were merged into 22 regions based on definitions from a previous analysis by the ADNI PiB PET Core [35]. Details regarding the process of merging the VOIs are presented in Table S3 of the Supplementary Materials. To examine spillover to non-brain tissues and air in the PVC, an additional VOI consisting of a 15 mm “shell” surrounding the outer surface of the brain was used. The VOIs for each participant were used for the calculation of GTM for PVC and subsequent VOI analysis. A VOI map for a representative case is shown in Figure S1.

### PoR framework

**Fig. 1:**
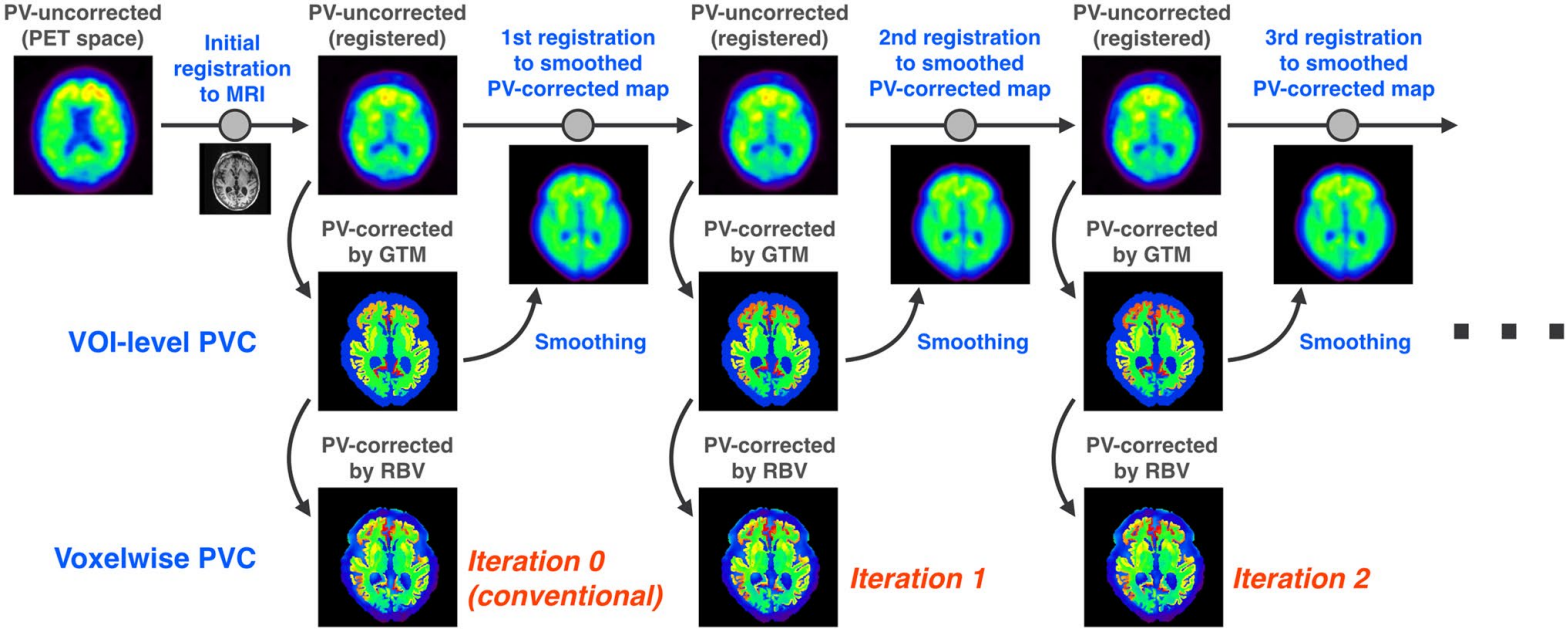
Flow of the PoR framework

The newly developed framework, PoR, iteratively performs MR-PVC and registration between the smoothed and PV-corrected map and the uncorrected PET image. Briefly, PoR is performed as follows (Fig. 1):

i. The uncorrected PET image was initially registered to an individual MR T1-weighted image. This step corresponds to the “conventional” registration method.
ii. The registered PET image is PV corrected using the GTM method [17], which generates a synthetic PET image containing PV-corrected radioactivity concentration values for each VOI.
iii. The synthetic PET image was smoothed using the PSF to match the resolution of the uncorrected PET image.
iv. The uncorrected PET image is registered to the smoothed synthetic PET image again.
v. Steps ii–iv are repeated until convergence.

The registration processes were performed with the normalized mutual information criteria [36] using the Statistical Parametric Mapping 12 package (https://www.fil.ion.ucl.ac.uk/spm/software/spm12). The GTM matrix was calculated once during the first iteration and then used for subsequent iterations. Smoothing and PVC were performed using custom-made Python (https://www.python.org) routines. To acquire a voxel-wise PV-corrected map, PVC with the region-based voxel-wise (RBV) method [19] is performed using the synthetic PET image in each iteration. Brief explanations for the GTM and RBV PVC methods are described in the Supplementary Materials. We assumed that the PET-MR misregistration in the initial registration process (step i) results in a discrepancy in uptake values between the initially registered and the smoothed synthetic PET images, calculated by steps ii and iii. The registration of the previously registered PET image to the smoothed synthetic PET image (step iv) attempts to reduce the discrepancy between the two images. Note that the smoothed synthetic PET image can be used as a target for registration to the MR geometric space, because the smoothed synthetic PET image has both MR anatomical information and a similar contrast to the original PET image. Step v, iteration of steps ii–iv, attempts to minimize the effect of the PET-MR misregistration on the GTM PVC process.

### Postprocessing of PV-uncorrected and PV-corrected PET images

Standardized uptake value (SUV) maps for the non-PV-corrected (PV-uncorrected) and PV-corrected PET images were calculated using the injected dose and body weight obtained from the ADNI database. Ratios of SUV to a reference region (i.e., cerebellar GM) were used to calculate the SUV ratio (SUVR).

### PoR versus conventional registration

To evaluate the performance of the PoR framework, we compared the results obtained from the PoR and the conventional registration. Differences in the estimates of translation and rotation between the conventional and PoR methods were calculated. Effect sizes of the differences in the estimates between the two methods were calculated as (mean of the difference)/(standard deviation of the difference). Correlation analysis between SUVR on the cerebral cortex and the differences in the registration estimates were performed to demonstrate the effect of tracer distribution on the registration error. The cortical SUVR in the correlation analysis was calculated by averaging PV-uncorrected SUVR on the anterior cingulate, frontal, parietal, and precuneus cortices, as previously reported [35].

The intra-region coefficient of variation (CoV; standard deviation/mean × 100%) of each VOI was calculated as an index to improve intra-region variability by PVC [19] and PoR. Differences in CoV were tested using the Wilcoxon signed-rank test to demonstrate the improvement of intra-region variability. The significance level for the tests was set at 0.05. The effect sizes of the differences in SUV, SUVR, and CoV between the two methods were calculated as the registration results.

## Results

### Trends of registration results estimated using PoR

**Fig. 2:**
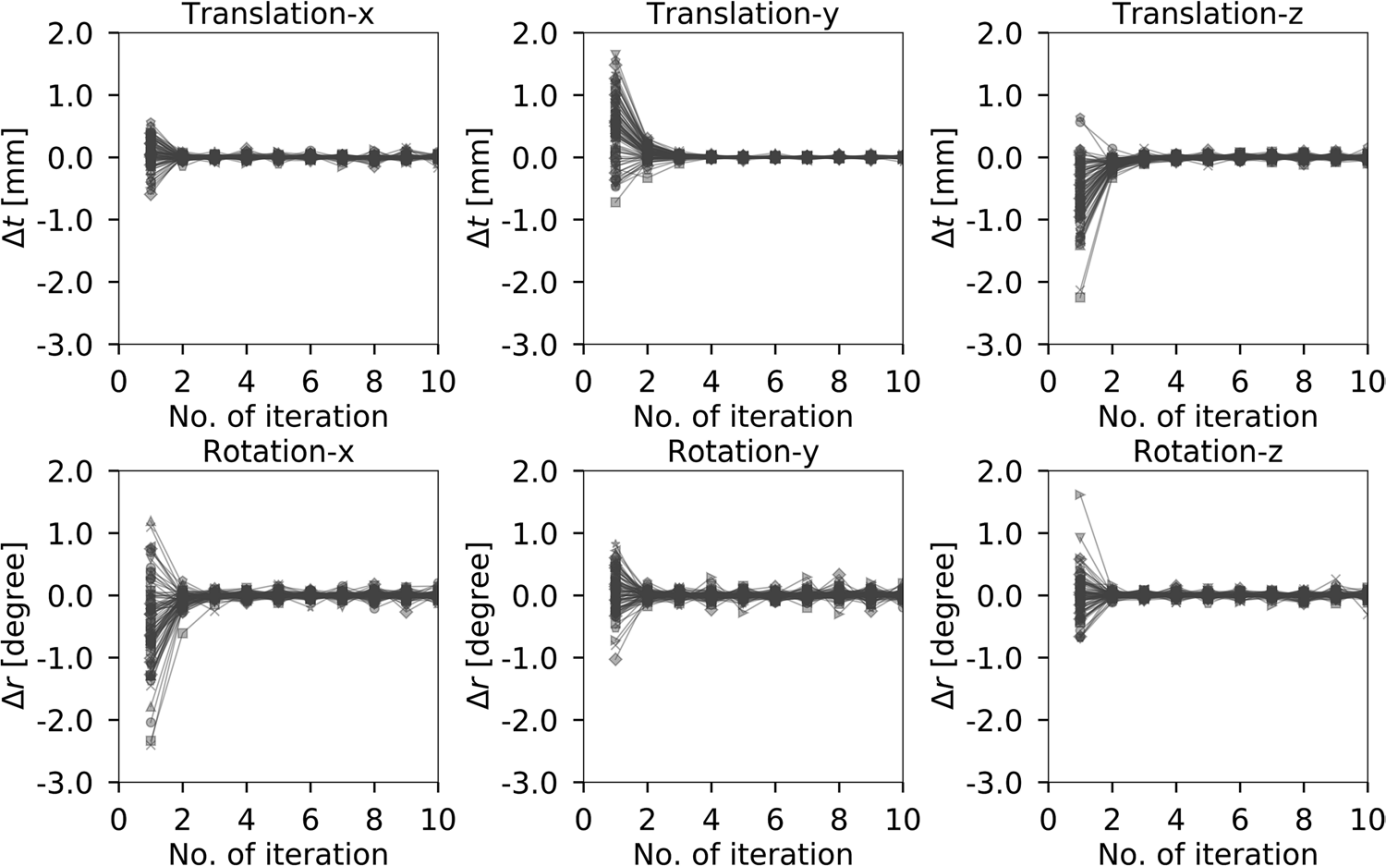
Trends in the difference in translations (Δ*t*) and rotations (Δ*r*) estimated by PoR from the previous iteration. Each line indicates the registration results for a single participant. Note that PoR converged after five iterations at most

The trends in the registration results estimated using PoR from the previous iteration are shown in Fig. 2. Registration using PoR converged after five iterations at most (Fig. 2). Therefore, for further analyses, the results for PoR using only five iterations are described herein.

### Differences in registration results between PoR and the conventional method

**Fig. 3:**
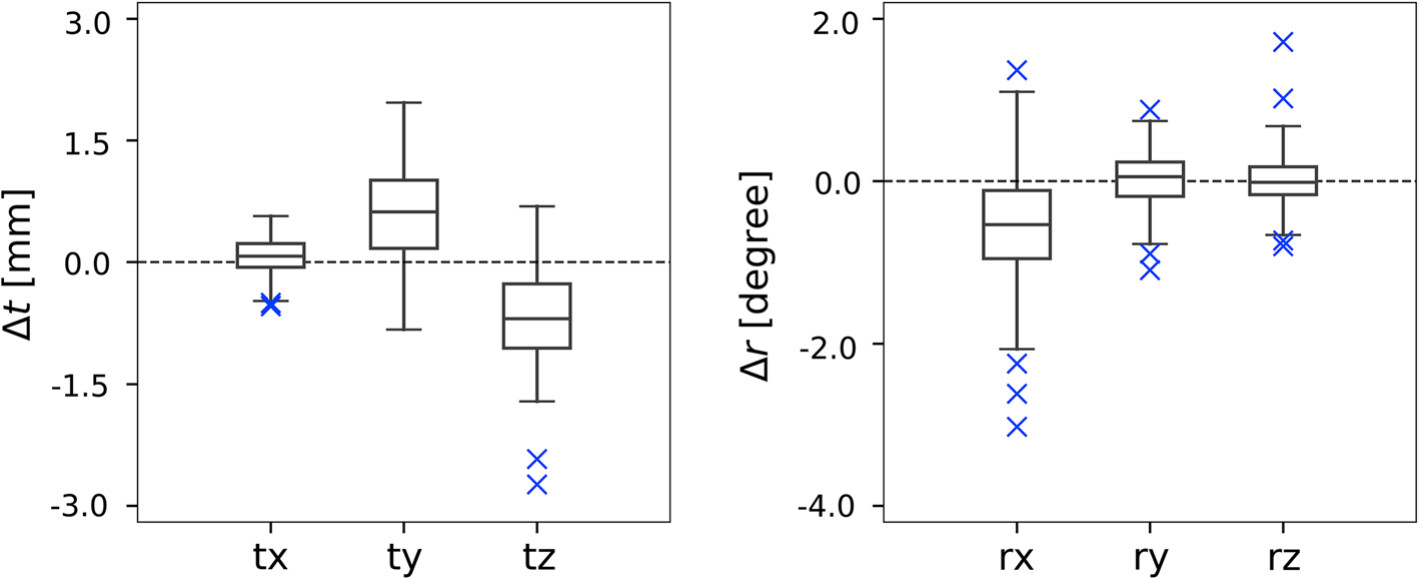
Differences in estimated translations and rotations between the conventional registration and PoR based on five iterations. Blue “ × ”s indicate outliers

The maximum differences between the two methods were 0.57, 1.97, and 2.74 mm for translations in the *x-*, *y-*, and *z*-axes, respectively, and 3.02°, 1.10°, and 1.72° for rotations in the *x*-, *y*-, and *z*-axes, respectively. The effect sizes of the differences in registration results were 0.26, 0.93, and − 1.22 for translations in the *x*-, *y*-, and *z*-axes, respectively, and − 0.72, 0.12, and 0.03 for rotations in the *x-*, *y-*, and *z*-axes, respectively. Considerable differences (effect size <  − 0.8 or effect size > 0.8) in translations on the y- and *z*-axes when applying PoR were observed, as shown in Fig. 3. Of the 92 participants, 5 (5.4%) showed a difference in translation greater than the voxel size for PET (2 mm).

**Fig. 4:**
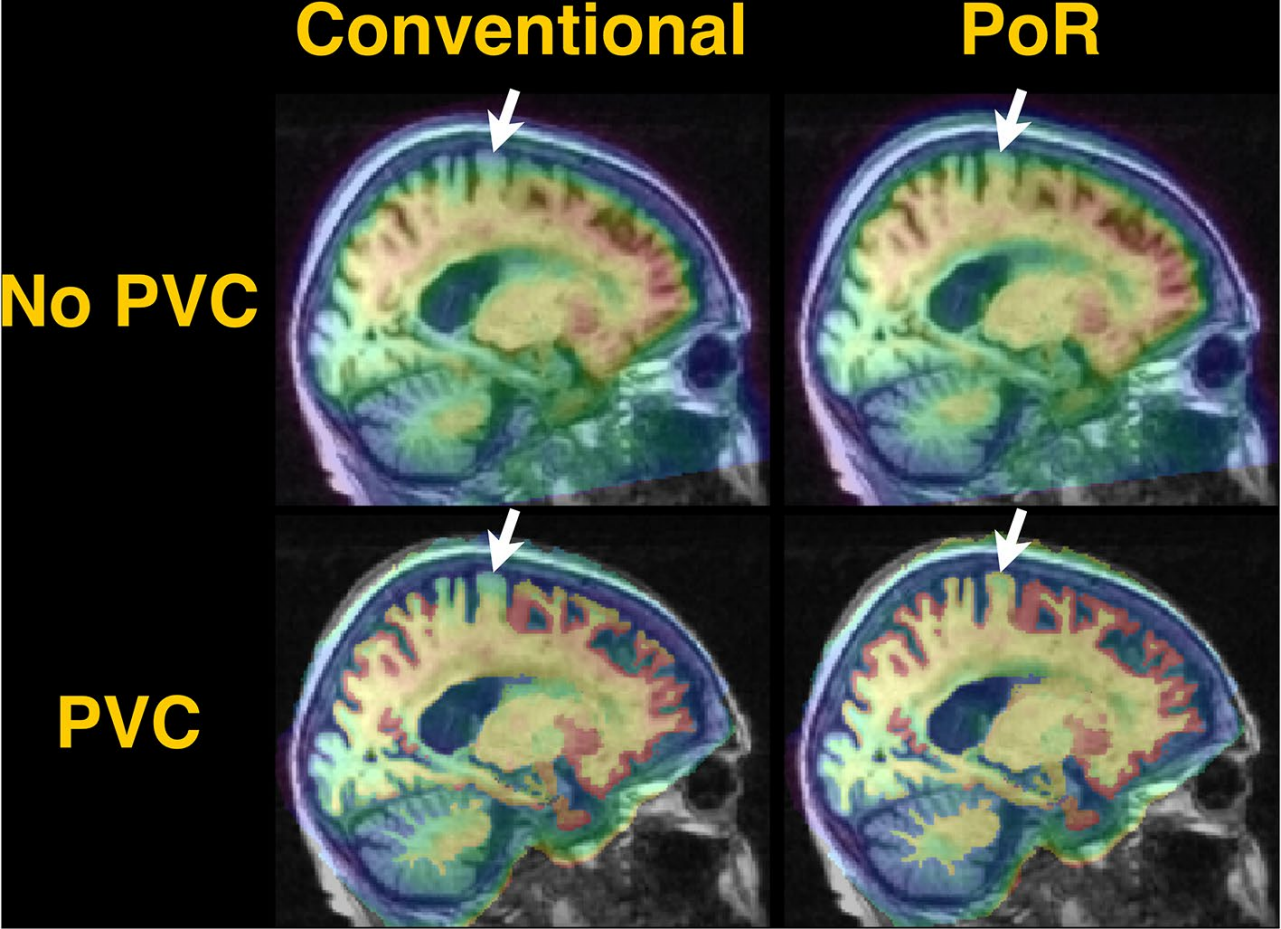
SUV images, fused with a MR T1-weighted image, without PVC (upper row) and with PVC (lower row) in a case with the largest translation on the *z*-axis (an 83-year-old female patient with MCI). Results are derived from using the conventional registration (left) or PoR (right). Thus, images on upper left, upper right, lower left and lower right indicate PV-uncorrected SUV maps registered with the conventional and PoR methods and PV-corrected SUV maps registered with the conventional and PoR methods, respectively. Differences between the two methods in this case were 0.32, 0.65, and 2.74 mm for translations in the *x-*, *y-*, and *z*-axes, respectively, and 3.02°, 0.34°, and 0.35° for rotations in the *x-*, *y-*, and *z*-axes, respectively. White arrows indicate parietal regions where remarkable differences in SUV between the conventional and PoR methods were observed

Figure 4 shows images from a case with the largest translation in the z-axis (2.74 mm). Extremely low SUV was observed in the parietal cortex owing to imprecise registration of the uncorrected PET image using the conventional method (white arrow in Fig. 4, upper left). This imprecise registration resulted in a non-uniform PV-corrected SUV in the parietal cortex (white arrow in Fig. 4, lower left). The low uptake in the parietal cortex was recovered using the PoR method, and apparent structure of parietal cortex was observed (white arrow in Fig. 4, upper right). Please see also zoomed SUV maps shown on Figure S2. Moreover, the uniformity of the PV-corrected SUV in the cerebellar GM was visibly improved by PoR. Similar trends were observed in other cases with large differences in translation in the *z*-axis (Figure S3).

A significant correlation between the cortical SUVR and the differences in rotation on the *x*-axis among the two registration methods was observed (*r* =  − 0.496; *p* < 0.001), as shown in Figure S4.

### Comparison of intra-region variability

**Fig. 5:**
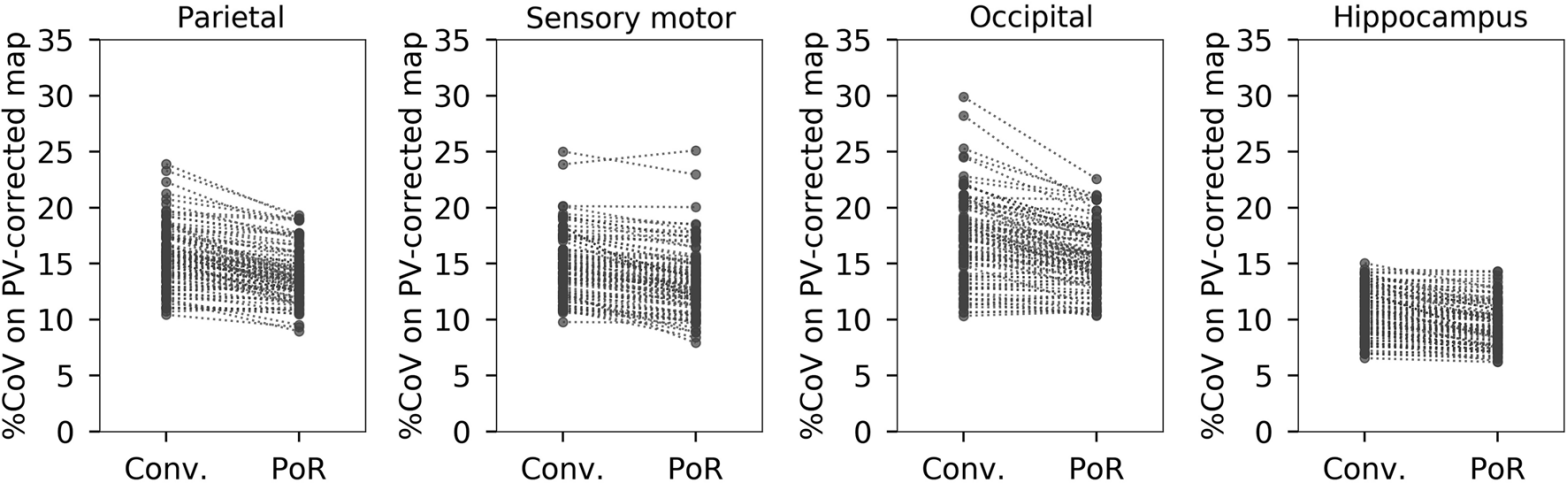
Changes in CoV on PV-corrected SUV maps between conventional registration and PoR in the VOIs in which considerable reduction was observed

The intra-region CoV of the PV-corrected SUV was significantly reduced by PoR throughout the following brain regions: frontal, parietal, occipital, medial temporal, sensory motor, and insula cortices, hippocampus, and cerebellum (*p* < 0.05, Table 1; Figure S5). Considerable reductions (effect size <  − 0.8) were observed in the parietal, occipital, and sensory motor cortices and hippocampus (Fig. 5; Table 1). Only the posterior cingulate cortex showed a significant increase in CoV. Significant decreases in the intra-region CoV using PVC were also observed in all regions of the brain (all *p* < 0.001, effect size <  − 1.2) (Tables S4 and S5). For example, CoV values in the region indicated by the white arrows in Fig. 4 (sensory motor cortex, see also Figure S2) in the case with the largest *z*-axis translation were as follows: 25.8% (PV uncorrected, conventional registration), 19.1% [PV uncorrected, PoR], 18.3% [PV corrected, conventional registration], and 11.7% [PV corrected, PoR].

**Table 1:** Values and their differences in intra-region CoV on PV-corrected SUV maps between conventional registration and PoR. Q1 and Q3 refer to the 1st and 3rd quantiles, respectively

VOI	Conventional [median (Q1, Q3)]	PoR [median (Q1, Q3)]	ΔCoV [median (Q1, Q3)]	*p* value	Effect size
Frontal cortex	13.6 (12.5, 15.8)	13.5 (11.7, 16.1)	− 0.4 (− 0.8, 0.4)	0.018	− 0.19
Parietal cortex	15.7 (13.9, 17.4)	13.6 (12.4, 15.3)	− 1.6 (− 2.6, 0.5)	< 0.001	− 1.27
Precuneus	10.6 (9.6, 11.9)	10.6 (9.2, 12.2)	− 0.1 (− 0.6, 0.4)	0.123	− 0.18
Occipital cortex	17.3 (14.8, 19.4)	15.1 (13.6, 17.2)	− 1.5 (− 3.2, − 0.4)	< 0.001	− 0.94
Lateral temporal cortex	14.8 (13.1, 17.3)	15.0 (12.9, 17.5)	0.2 (− 0.2, 0.5)	0.079	0.12
Medial temporal cortex	13.2 (11.4, 16.1)	12.9 (11.2, 15.2)	− 0.3 (− 1.4, 0.3)	< 0.001	− 0.46
Sensory motor cortex	14.4 (12.5, 16.2)	13.2 (11.4, 14.9)	− 1.2 (− 2.0, − 0.5)	< 0.001	− 1.15
Anterior cingulate cortex	9.1 (7.7, 10.6)	8.9 (7.8, 10.4)	− 0.1 (− 0.4, 0.3)	0.115	− 0.16
Posterior cingulate cortex	10.4 (9.0, 11.8)	11.2 (9.7, 12.5)	0.5 (0.0, 1.2)	< 0.001	0.56
Insula	9.9 (8.9, 11.5)	9.6 (8.9, 11.5)	− 0.1 (− 0.3, 0.1)	< 0.001	− 0.43
Hippocampus	10.5 (8.9, 12.3)	9.4 (8.1, 10.7)	− 0.8 (− 1.5, − 0.3)	< 0.001	− 1.02
Cerebellum	16.5 (14.1, 18.0)	15.0 (13.1, 16.9)	− 0.8 (− 1.6, 0.0)	< 0.001	− 0.57

*CoV* coefficient of variation, *PV* partial volume, *SUV* standardized uptake value, *VOI* volume of interest

### Comparison of SUV and SUVR between PoR and the conventional method

**Fig. 6:**
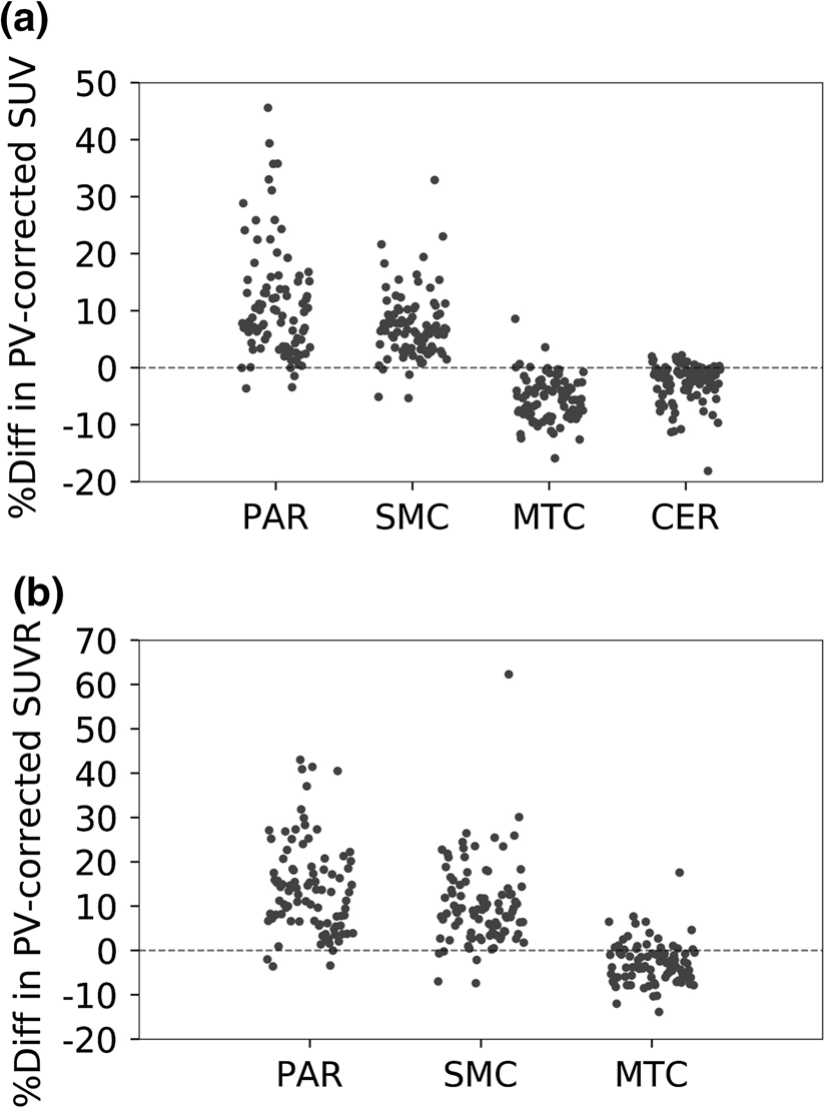
Percentage differences in PV-corrected SUV (**a**, top) and SUVR (**b**, bottom) between conventional registration and PoR for VOIs at the top (parietal and sensory motor cortices) and bottom (medial temporal cortex and cerebellum) of the brain. PAR, SMC, MTC and CER indicate the parietal cortex, sensory motor cortex, medial temporal cortex and cerebellum, respectively

Considerable increases (effect size of approximately 0.8) in PV-corrected SUV between PoR and the conventional method were observed with regions at the top of the brain (parietal and sensory motor cortices), whereas considerable decreases (effect size of approximately − 0.8) were observed at the bottom of the brain (temporal cortex and insula) (Figs. 6a, S7; Table S6). Moreover, the SUV in the cerebellum moderately decreased when PoR was applied (effect size: − 0.62).

On applying PoR, PV-corrected SUVR increased in all regions (effect size > 0; Figs. 6b, S8; Table S7), except in the medial temporal cortex. Significant differences (effect size > 0.8) were observed in the parietal and sensory motor cortices. A maximal difference of 62.3% was observed in the sensory motor cortex.

## Discussion

Differences in the results between the conventional registration and PoR were smaller than the voxel size used in PET (2 mm) in most participants. However, these small differences, particularly the translation in the *z*-axis and rotation in the *x*-axis, resulted in significant differences in PV-corrected SUV in the upper and lower brain regions. The biases in SUV in the cerebellum, located at the bottom of the brain, thus caused an underestimation of SUVR throughout the brain. These results were consistent with a previous systematic study that reported high bias in PV-corrected radioactivity concentration using GTM due to registration error [28]. These findings suggest that even small shifts in registration at a sub-voxel level affect the accuracy of quantification using PVC; therefore, precise registration is required for accurate PVC.

Registrations between PET and MR spaces obtained using the novel PoR framework were visibly more consistent than those obtained using the conventional registration. For example, low values on uncorrected and PV-corrected SUV maps of the parietal region were observed even in suspected amyloid-positive cases, representing cases of erroneous axial shift with the conventional registration. In the PoR framework, uncorrected and PV-corrected SUV in the parietal region were consistent with the surrounding regions.

The PoR framework resulted in lower intra-region variability in the PV-corrected SUV than with the conventional registration. The intra-region variability, measured as the CoV in each region, can be affected by spillover between the target and the surrounding regions due to the partial volume effect. Thus, intra-region CoV has been used as an index for PVC to perform appropriately. For example, Thomas et al. [19] reported that PVC using the RBV method reduced intra-region variability, and they suggested that RBV does not significantly amplify noise but reduces variability in the cortex. The intra-region variability on the PV-corrected map is also affected by the discrepancy in position between VOIs and PET images due to PET-MR misregistration. The reduction in intra-region variability in the present study reflects an improvement in consistency between PET and MR images as well as correcting spillover between the target and the surrounding regions by PVC. This finding suggests that PoR reduces the error in MR-PVC owing to imprecise registration.

The significant inter-method differences in PV-corrected SUV between the conventional registration and PoR could be associated with the improvement in PET-MR consistency by PoR. For example, in the case shown in Fig. 4, unnaturally low PV-corrected SUV in the parietal cortex, observed by the conventional registration, was recovered by PoR. These findings suggest that PoR can improve the accuracy of the quantification of amyloid using MR-PVC by improving PET-MR consistency. Further studies are required to validate the effect of the PoR framework on cross-sectional and longitudinal analyses.

Different tracer distributions can result in different PET-MR registration errors. A previous report demonstrated that the frequency of PET-MR registration errors increased with an increase in amyloid deposition [37]. Actually, in the present study, the larger cortical SUVR resulted in larger differences in rotation on the x-axis between the conventional registration and the PoR framework. Theoretically, the performance of the PoR framework is not affected by varying the tracer distribution and thus contrasts between PET and MR images because of the use of the smoothed synthetic image, which has MR anatomical information and contrast similar to that of the original PET image. Therefore, the PoR framework could be used for brain PET studies with other ^18^F-labeled ligands for amyloid imaging [3, 38, 39] and with radiotracers for other targets as well as PiB PET studies. In particular, PoR could be useful in cases in which the registration between PET and MR images is challenging because of different contrasts between these images, such as in dopamine transporter imaging [40, 41].

Considerable larger differences in the translation on the *y*- and *z*-axes between the conventional registration and PoR than in the other directions could be due to the asymmetric shape of the brain on the *y*- and *z*-axes. Generally, the brain is roughly symmetric on the *x*-axis, not on the *y*- and *z*-axes. No significant difference in the translation on the *x*-axis was observed in the present study. The trend in the mass of registration errors could change in the case of the other target organs.

Registration using PoR assumes that the PVC in the previous step is appropriately performed. Therefore, other error sources in MR-PVC, apart from the registration error, could affect the results of the PoR. In this study, we observed a significant increase in CoV in the posterior cingulate following PoR, which was contrary to the effect in other regions. We hypothesized that the poorer intra-region variability in the posterior cingulate resulted from a segmentation error in this region. In our experience, segmentation of the posterior cingulate could be more challenging than that of other regions because of its small size and the difficulty in distinguishing it from adjacent regions. Further systematic studies are required to investigate the effects of segmentation errors on the results of the PoR framework. Therefore, frameworks that can compensate for other error sources in MR-PVC, such as segmentation error, are required to improve the accuracy of MR-PVC.

Another limitation of this study is the fact that the utility of the PoR method was demonstrated by an improvement only in intra-region variability. One possible way of demonstrating the utility of the PoR method is to use numerical simulations in which the true motion can be known. However, simulating misregistration using conventional registration is very difficult because the causes of misregistration are unknown. Another method for demonstrating the same is through visual assessment by experts in neuroradiology. However, the assessment of sub-voxel differences, such as those observed in this study, is also very difficult, even for expert neuroradiogists. Therefore, we regarded intra-region variability as the only index for improving registration.

Mismatches between the attenuation map and emission data can result in large CoV [42] as well as misregistration of PET and anatomical images. Intra-frame motion can affect intra-region variability. However, attenuation maps and raw data were not available. Thus, motion correction for the attenuation–emission mismatch and intra-frame motion was not performed in this study. Further studies are required to demonstrate the effect of motion artifacts on PoR.

## Conclusion

We developed a novel framework (PoR) for image registration and PVC and applied it to the [^11^C]PiB PET dataset. Our results revealed that a registration error could result in under- and overestimation in PV-corrected SUV, despite the registration error being small. Moreover, the results indicated that the use of PoR prevented imprecise registration and reduced the associated biases in the PV-corrected SUV and SUVR. Furthermore, PoR improved the consistency between PET and MR images and reduced the intra-region variability of PET images. These findings suggest that PoR improves quantitative accuracy in brain PET studies as well as amyloid PET.

## Electronic supplementary material

Below is the link to the electronic supplementary material.

Supplementary file1 (PDF 3432 kb)

## References

[1] Klunk WE, Engler H, Nordberg A, Wang Y, Blomqvist G, Holt DP (2004). Imaging brain amyloid in Alzheimer’s disease with Pittsburgh Compound-B. Ann Neurol.

[2] Mathis CA, Wang Y, Holt DP, Huang G-F, Debnath ML, Klunk WE (2003). Synthesis and evaluation of ^11^C-labeled 6-substituted 2-arylbenzothiazoles as amyloid imaging agents. J Med Chem.

[3] Nelissen N, Laere KV, Thurfjell L, Owenius R, Vandenbulcke M, Koole M (2009). Phase 1 study of the pittsburgh compound b derivative ^18^F-flutemetamol in healthy volunteers and patients with probable Alzheimer disease. J Nucl Med.

[4] Vandenberghe R, Van Laere K, Ivanoiu A, Salmon E, Bastin C, Triau E (2010). ^18^F-flutemetamol amyloid imaging in Alzheimer disease and mild cognitive impairment: a phase 2 trial. Ann Neurol.

[5] Chien DT, Bahri S, Szardenings AK, Walsh JC, Mu F, Su M-Y (2013). Early clinical pet imaging results with the novel phf-tau radioligand [F-18]-T807. J Alzheimers Dis.

[6] Harada R, Okamura N, Furumoto S, Furukawa K, Ishiki A, Tomita N (2016). ^18^F-THK5351: a novel pet radiotracer for imaging neurofibrillary pathology in Alzheimer disease. J Nucl Med.

[7] Maruyama M, Shimada H, Suhara T, Shinotoh H, Ji B, Maeda J (2013). Imaging of tau pathology in a tauopathy mouse model and in Alzheimer patients compared to normal controls. Neuron.

[8] Okamura N, Furumoto S, Harada R, Tago T, Yoshikawa T, Fodero-Tavoletti M (2013). Novel ^18^F-labeled arylquinoline derivatives for noninvasive imaging of tau pathology in Alzheimer disease. J Nucl Med.

[9] Hoffman EJ, Huang S-C, Phelps ME (1979). Quantitation in positron emission computed tomography: 1. effect of object size. J Comput Assist Tomogr..

[10] Agdeppa ED, Kepe V, Liu J, Flores-Torres S, Satyamurthy N, Petric A (2001). Binding characteristics of radiofluorinated 6-dialkylamino-2-naphthylethylidene derivatives as positron emission tomography imaging probes for β-amyloid plaques in Alzheimer’s disease. J Neurosci..

[11] Matsubara K, Ibaraki M, Shimada H, Ikoma Y, Suhara T, Kinoshita T (2016). Impact of spillover from white matter by partial volume effect on quantification of amyloid deposition with [^11^C]PiB PET. NeuroImage.

[12] Alessio AM, Kinahan PE (2006). Improved quantitation for PET/CT image reconstruction with system modeling and anatomical priors. Med Phys.

[13] Baete K, Nuyts J, Laere KV, Van Paesschen W, Ceyssens S, De Ceuninck L (2004). Evaluation of anatomy based reconstruction for partial volume correction in brain FDG-PET. NeuroImage.

[14] Erlandsson K, Dickson J, Arridge S, Atkinson D, Ourselin S, Hutton BF (2016). MR imaging-guided partial volume correction of PET data in PET/MR imaging. PET Clin.

[15] Meltzer CC, Leal JP, Mayberg HS, Wagner HN, Frost JJ (1990). Correction of PET data for partial volume effects in human cerebral cortex by MR imaging. J Comput Assist Tomogr.

[16] Müller-Gärtner HW, Links JM, Prince JL, Bryan RN, McVeigh E, Leal JP (1992). Measurement of radiotracer concentration in brain gray matter using positron emission tomography: MRI-based correction for partial volume effects. J Cereb Blood Flow Metab.

[17] Rousset OG, Ma Y, Evans AC (1998). Correction for partial volume effects in PET: principle and validation. J Nucl Med.

[18] Shidahara M, Tsoumpas C, Hammers A, Boussion N, Visvikis D, Suhara T (2009). Functional and structural synergy for resolution recovery and partial volume correction in brain PET. NeuroImage.

[19] Thomas BA, Erlandsson K, Modat M, Thurfjell L, Vandenberghe R, Ourselin S (2011). The importance of appropriate partial volume correction for PET quantification in Alzheimer’s disease. Eur J Nucl Med Mol Imaging.

[20] Arakawa R, Stenkrona P, Takano A, Nag S, Maior RS, Halldin C (2017). Test-retest reproducibility of [^11^C]-l-deprenyl-d2 binding to MAO-B in the human brain. EJNMMI Res.

[21] Brendel M, Högenauer M, Delker A, Sauerbeck J, Bartenstein P, Seibyl J (2015). Improved longitudinal [^18^F]-AV45 amyloid PET by white matter reference and VOI-based partial volume effect correction. NeuroImage.

[22] Habert M-O, Bertin H, Labit M, Diallo M, Marie S, Martineau K (2018). Evaluation of amyloid status in a cohort of elderly individuals with memory complaints: validation of the method of quantification and determination of positivity thresholds. Ann Nucl Med.

[23] LaPoint MR, Chhatwal JP, Sepulcre J, Johnson KA, Sperling RA, Schultz AP (2017). The association between tau PET and retrospective cortical thinning in clinically normal elderly. NeuroImage.

[24] Schaeverbeke J, Evenepoel C, Declercq L, Gabel S, Meersmans K, Bruffaerts R (2018). Distinct [^18^F]THK5351 binding patterns in primary progressive aphasia variants. Eur J Nucl Med Mol Imaging..

[25] Strul D, Bendriem B (1999). Robustness of anatomically guided pixel-by-pixel algorithms for partial volume effect correction in positron emission tomography. J Cereb Blood Flow Metab.

[26] Yang J, Huang SC, Mega M, Lin KP, Toga AW, Small GW (1996). Investigation of partial volume correction methods for brain FDG PET studies. IEEE Trans Nucl Sci.

[27] Frouin V, Comtat C, Reilhac A, Grégoire M-C (2002). Correction of partial-volume effect for PET striatal imaging: fast implementation and study of robustness. J Nucl Med.

[28] Sattarivand M, Kusano M, Poon I, Caldwell C (2012). Symmetric geometric transfer matrix partial volume correction for PET imaging: principle, validation and robustness. Phys Med Biol.

[29] Rullmann M, Dukart J, Hoffmann K-T, Luthardt J, Tiepolt S, Patt M (2016). Partial-volume effect correction improves quantitative analysis of 18F-florbetaben β-amyloid PET scans. J Nucl Med.

[30] Su Y, Blazey TM, Snyder AZ, Raichle ME, Marcus DS, Ances BM (2015). Partial volume correction in quantitative amyloid imaging. NeuroImage.

[31] Schwarz CG, Gunter JL, Lowe VJ, Weigand S, Vemuri P, Senjem ML, et al. A comparison of partial volume correction techniques for measuring change in serial amyloid PET SUVR. J Alzheimers Dis. 2018;Preprint:1–15 10.3233/JAD-180749 PMC6398556 30475770

[32] Fischl B, van der Kouwe A, Destrieux C, Halgren E, Ségonne F, Salat DH (2004). Automatically parcellating the human cerebral cortex. Cereb Cortex.

[33] Fischl B, Salat DH, Busa E, Albert M, Dieterich M, Haselgrove C (2002). Whole brain segmentation: automated labeling of neuroanatomical structures in the human brain. Neuron.

[34] Desikan RS, Ségonne F, Fischl B, Quinn BT, Dickerson BC, Blacker D (2006). An automated labeling system for subdividing the human cerebral cortex on MRI scans into gyral based regions of interest. NeuroImage.

[35] Jagust WJ, Bandy D, Chen K, Foster NL, Landau SM, Mathis CA (2010). The ADNI PET core. Alzheimers Dement J Alzheimers Assoc.

[36] Studholme C, Hawkes DJ, Hill DL. Normalized entropy measure for multimodality image alignment. 1998; http://dx.doi.org/10.1117/12.310835

[37] Schwarz CG, Jones DT, Gunter JL, Lowe VJ, Vemuri P, Senjem ML (2017). Contributions of imprecision in PET-MRI rigid registration to imprecision in amyloid PETSUVR measurements. Hum Brain Mapp.

[38] Barthel H, Gertz H-J, Dresel S, Peters O, Bartenstein P, Buerger K (2011). Cerebral amyloid-β PET with florbetaben (^18^F) in patients with Alzheimer’s disease and healthy controls: a multicentre phase 2 diagnostic study. Lancet Neurol.

[39] Carome M, Wolfe S (2011). Florbetapir-pet imaging and postmortem β-amyloid pathology. JAMA.

[40] Müller U, Wächter T, Barthel H, Reuter M, von Cramon DY (2000). Striatal [123I]β-CIT SPECT and prefrontal cognitive functions in Parkinson’s disease. J Neural Transm.

[41] Rinne JO, Sahlberg N, Ruottinen H, Någren K, Lehikoinen P (1998). Striatal uptake of the dopamine reuptake ligand [11C]beta-CFT is reduced in Alzheimer’s disease assessed by positron emission tomography. Neurology.

[42] Wardak M, Wong K-P, Shao W, Dahlbom M, Kepe V, Satyamurthy N, Small GW, Barrio JR, Huang S-C (2010). Movement correction method for human brain pet images: application to quantitative analysis of dynamic 18F-FDDNP scans. J Nucl Med.

