# Supplementary material for: Iterative framework for image registration and partial volume correction in brain positron emission tomography
Source: Radiol Phys Technol. 2020 Oct 19;13(4):348–57. doi: 10.1007/s12194-020-00591-2 (PMC7688593; doi:10.1007/s12194-020-00591-2)
Supplement: Supplementary file 1 — Supplementary file1 (PDF 3432 kb) [file 12194_2020_591_MOESM1_ESM.pdf]

**Supplementary Materials**

**Brief summary for partial volume correction (PVC) methods**

*Geometric transfer matrix (GTM)*

The GTM method [17] is a region-based MR-PVC algorithm using VOIs extracted with MR segmentation algorithm. In the GTM method, spillover between target and its surrounding regions are computed with VOIs and point spread function (PSF). This matrix is called with 'geometric transfer matrix (GTM)'. Then, true activities on VOIs are estimated from the GTM and measured activities averaged on each VOI as follows:

$$T = G^{-1} \cdot r$$

where $T$ and $r$ indicate the n × 1 vectors containing true and measured activities on VOIs, respectively; $G$ indicates the GTM, n × n matrix containing $g_{ij}$, which indicates the fractional contribution of activity from region $j$ to region $i$; and n indicates number of VOIs. Note that the GTM method assumes homogeneous activity on each VOI.

For voxelwise PVC with region-based voxelwise (RBV) algorithm and iterative registration processes in the PoR method, the synthetic PET image ($s(x)$) is calculated as follows:

$$s(x) = \sum_{i=1,\dots,n} [T_i \cdot p_i(x)]$$

where $x$ indicates position of each voxel; $T_i$ indicates the true activity on region $i$, estimated with the GTM method; and $p_i(x)$ is 1 if $x$ is inside region $i$, otherwise 0.

*Region-based voxelwise (RBV)*

The RBV algorithm [19] is an extension method for the GTM method to perform voxelwise PVC. In the RBV algorithm, voxelwise PV-corrected map ($f_c(x)$) is computed with the measured and synthetic PET images as follows:

$$f_c(x) = f(x) \cdot \left[\frac{s(x)}{s(x) \otimes h(x)}\right]$$

where $f(x)$ indicates measured PET image, and $h(x)$ indicates PSF.

**Figures and Tables**

Table S1. **List of image and voxel sizes for the downloaded original PET images in the present study.**

| | | No. of | Image size | | | Voxel size [mm] | | |
|---|---|---|---|---|---|---|---|---|
| Manufacturer | PET model | subjects | X | Y | Z | X | Y | Z |
| Siemens | ECAT Exact HR+ | 30 | 128 | 128 | 63 | 2.06 | 2.06 | 2.06 |
| | | 5 | 128 | 128 | 63 | 2.25 | 2.25 | 2.25 |
| | | 6 | 128 | 128 | 63 | 2.57 | 2.57 | 2.57 |
| | HRRT | 23 | 256 | 256 | 256 | 1.22 | 1.22 | 1.22 |
| GE | Advance | 21 | 128 | 128 | 35 | 2.00 | 2.00 | 4.25 |
| | Discovery RX | 7 | 128 | 128 | 47 | 2.00 | 2.00 | 3.27 |

Table S2. **Sizes of Gaussian kernel applied to the downloaded original PET images in the present study.**

| Manufacturer | PET model | No. of subjects | X | Y | Z |
|---|---|---|---|---|---|
| Siemens | ECAT Exact HR+ | 41 | 5.0 | 5.0 | 5.0 |
| | HRRT | 23 | 6.0 | 6.0 | 6.0 |
| GE | Advance | 21 | 5.0 | 5.0 | 2.0 |
| | Discovery RX | 7 | 5.0 | 5.0 | 3.5 |

Unit for X, Y and Z: mm at full width half-maximum (FWHM)

Table S3. **List of FreeSurfer parcellation regions merged into each VOI in the present study.**

| VOI | FreeSurfer parcellation |
|---|---|
| Frontal cortex | ctx-lh(rh)-caudalmiddlefrontal |
| | ctx-lh(rh)-lateralorbitofrontal |
| | ctx-lh(rh)-medialorbitofrontal |
| | ctx-lh(rh)-parsopercularis |
| | ctx-lh(rh)-parsorbitalis |
| | ctx-lh(rh)-parstriangularis |
| | ctx-lh(rh)-rostralmiddlefrontal |
| | ctx-lh(rh)-superiorfrontal |
| | ctx-lh(rh)-frontalpole |
| Parietal cortex | ctx-lh(rh)-inferiorparietal |

| | |
|---|---|
| | ctx-lh(rh)-superiorparietal |
| | ctx-lh(rh)-supramarginal |
| Precuneus | ctx-lh(rh)-precuneus |
| Occipital cortex | ctx-lh(rh)-cuneus |
| | ctx-lh(rh)-lateraloccipital |
| | ctx-lh(rh)-lingual |
| | ctx-lh(rh)-pericalcarine |
| Lateral temporal cortex | ctx-lh(rh)-bankssts |
| | ctx-lh(rh)-inferiortemporal |
| | ctx-lh(rh)-middletemporal |
| | ctx-lh(rh)-superiortemporal |
| | ctx-lh(rh)-temporalpole |
| | ctx-lh(rh)-transversetemporal |
| Medial temporal cortex | ctx-lh(rh)-enthorhinal |
| | ctx-lh(rh)-fusiform |
| | ctx-lh(rh)-parahippocampal |
| Anterior cingulate cortex | ctx-lh(rh)-caudalanteriorcingulate |
| | ctx-lh(rh)-rostralanteriorcingulate |
| Posterior cingulate cortex | ctx-lh(rh)-isthmuscingulate |
| | ctx-lh(rh)-posteriorcingulate |
| Sensory motor cortex | ctx-lh(rh)-paracentral |
| | ctx-lh(rh)-postcentral |
| | ctx-lh(rh)-precentral |
| Insula | ctx-lh(rh)-insula |
| Caudate | Caudate |
| Putamen | Putamen |
| Thalamus | Thalamus-Proper |
| | Thalamus |
| | VentralDC |
| Accumbens area | Accumbens area |
| Pallidum | Pallidum |
| Brainstem | Brainstem |

| | |
|---|---|
| Hippocampus | Hippocampus |
| Amygdala | Amygdala |
| Cerebellar gray matter | Cerebellum–Cortex |
| Subcortical white matter | Cerebral white matter |
| | WM hypointensities |
| | CC_Posterior |
| | CC_Mid_Posterior |
| | CC_Central |
| | CC_Mid_Anterior |
| | CC_Anterior |
| Cerebellar white matter | Cerebellum–white matter |
| Cerebrospinal fluid | Lateral ventricle |
| | Inf–Lat–Vent |
| | 3rd ventricle |
| | 4th ventricle |
| | CSF |
| | vessel |
| | choroid plexus |
| | 5th ventricle |

The 113 regions parcellated using FreeSurfer (aparc + aseg) were merged into 22 regions for region-based PVC and VOI analysis in this study. MR image and VOI map for a representative case are shown in Figure S1.

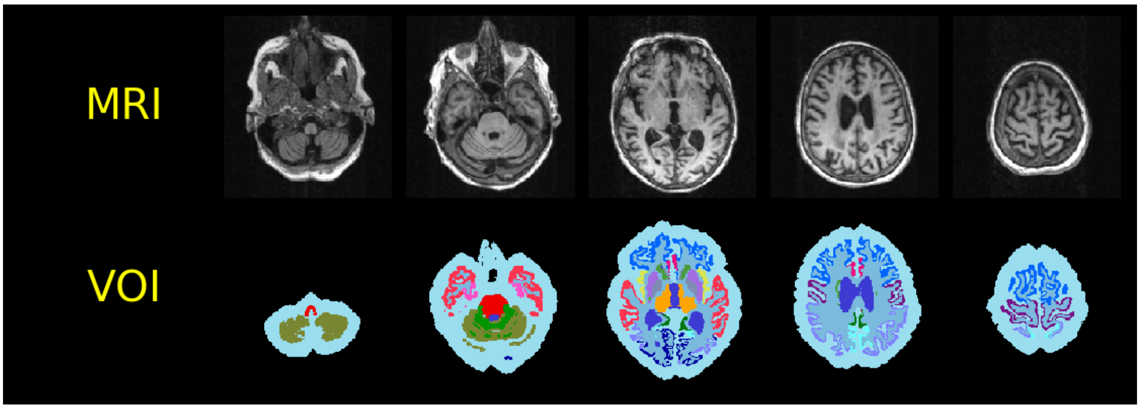

Figure S1 MR image and VOI map for a representative case.

Figure S2    VOI and SUV maps zoomed to the sensory motor cortex in the same case shown in Figure 4. Note that the colormaps for the SUV maps differ from those in Figure 4 because different software was used to show maps to view VOI contours.

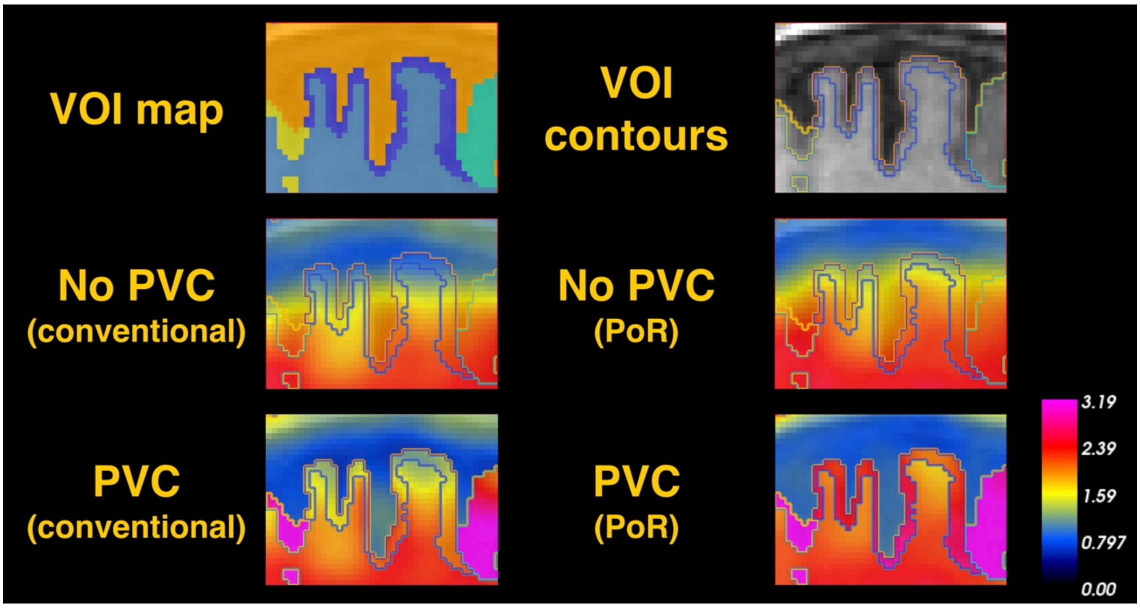

Figure S3.**Comparison of SUV images between conventional registration and PoR methods**

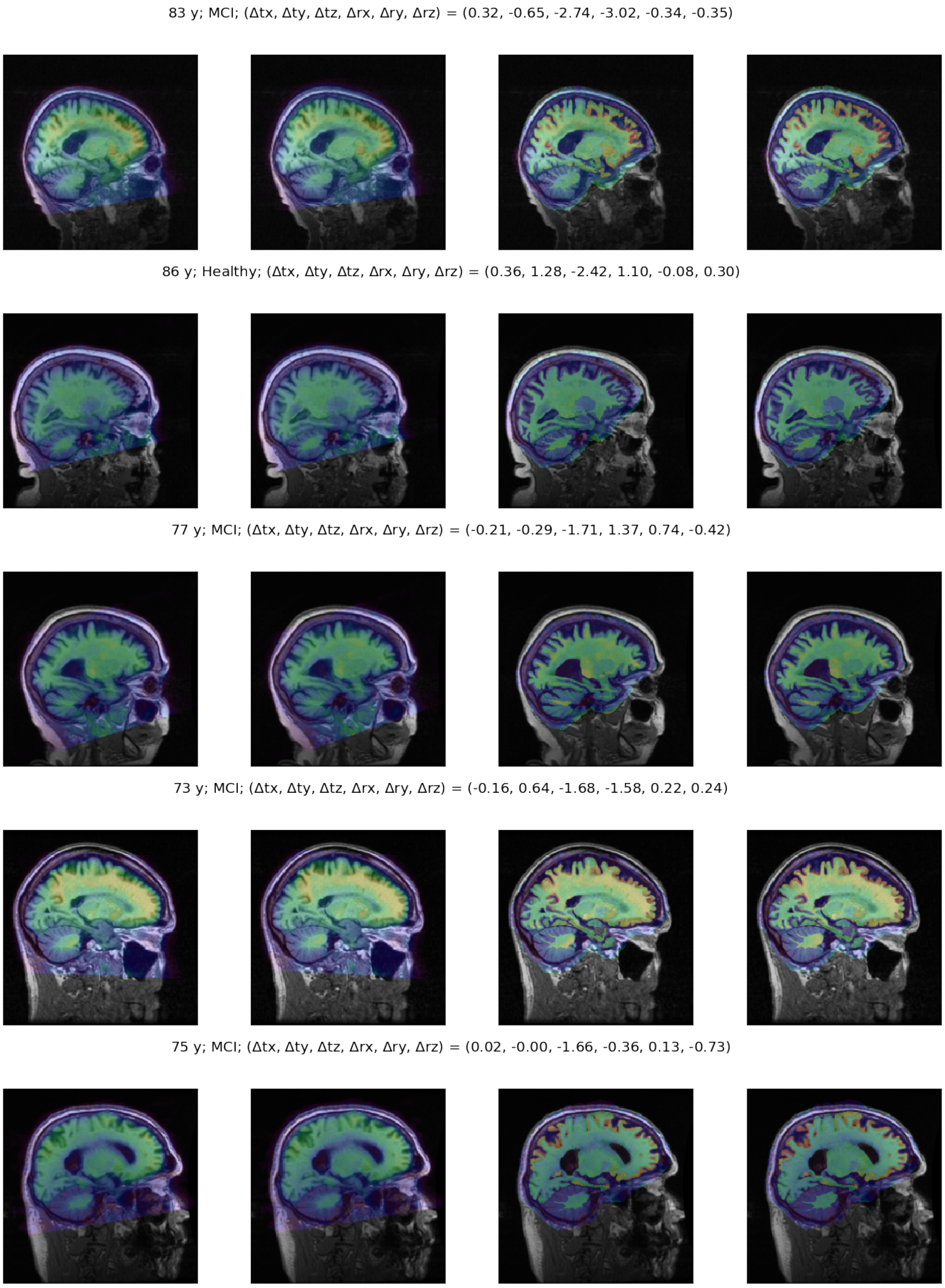


SUV images without PVC ($1^{st}$ and $2^{nd}$ columns) and with PVC ($3^{rd}$ and $4^{th}$ columns) for the five cases with the largest differences in translation in the z-axis between conventional registration ($1^{st}$ and $3^{rd}$ columns) and PoR ($2^{nd}$ and $4^{th}$ columns). Each row indicates images for a single participant. PVC, partial volume correction; SUV, standardized uptake value

Figure S4. **Scatter plots between uncorrected SUVR on the cerebral cortex and differences in the registration results between the conventional registration and PoR based on five iterations. The red line indicates a regression line in case that correlation was significant (*p* < 0.05).**

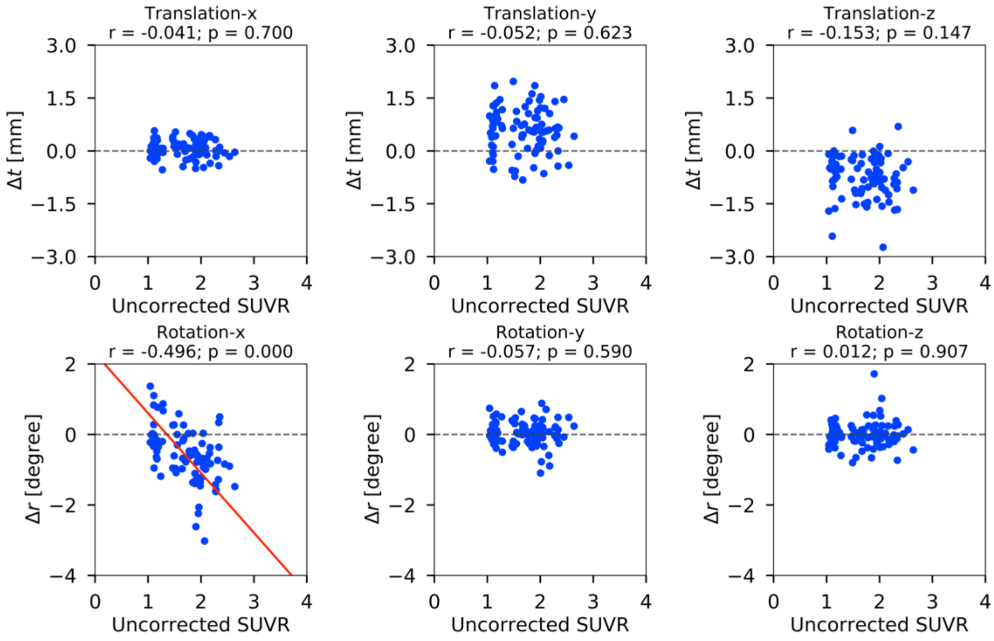

Figure S5. **Changes in CoV on PV-corrected SUV maps between conventional registration and PoR for all VOIs.**

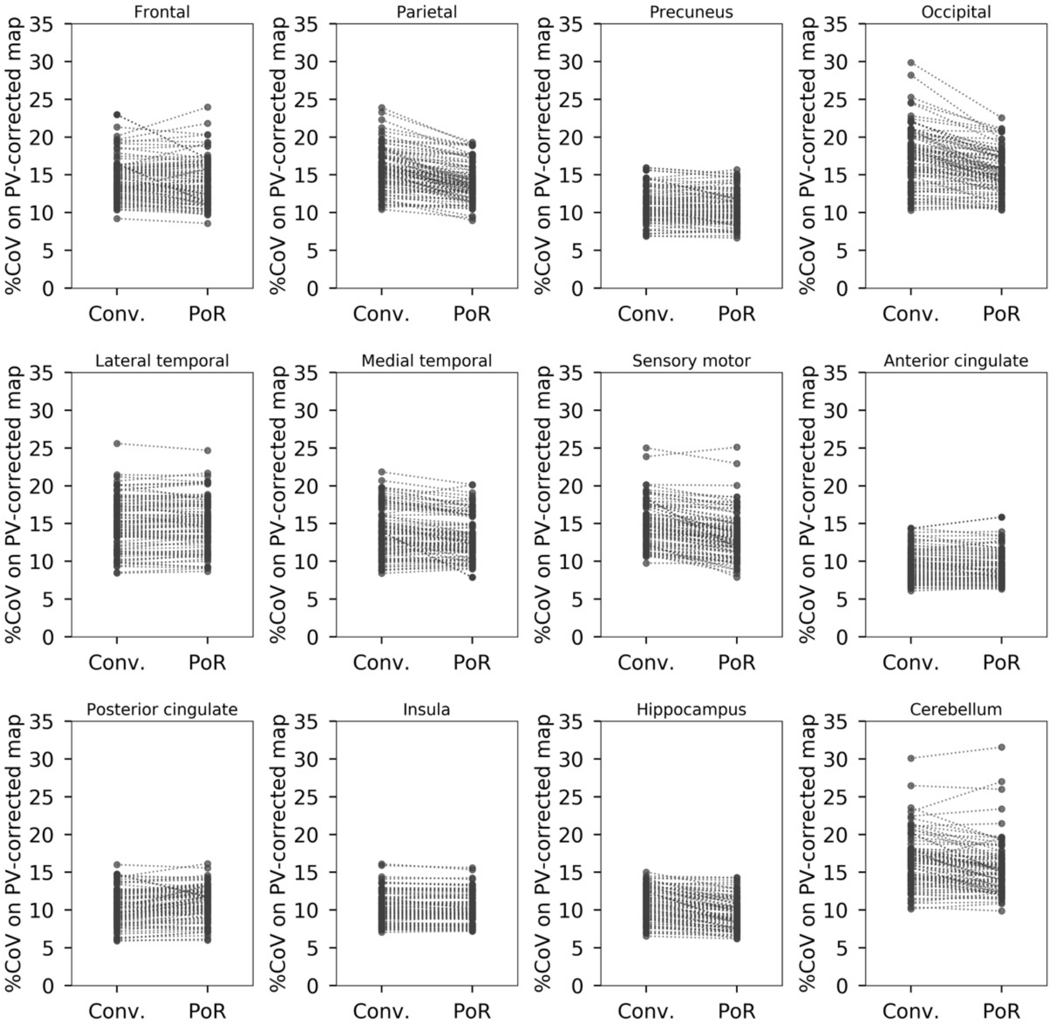


CoV, coefficient of variation; PoR, PVC-optimized registration; PV, partial volume; SUV, standardized uptake value; VOI, volume of interest.

Figure S6. **Changes in CoV between PV-uncorrected and PV-corrected SUV maps coregistered by PoR for all VOIs.**

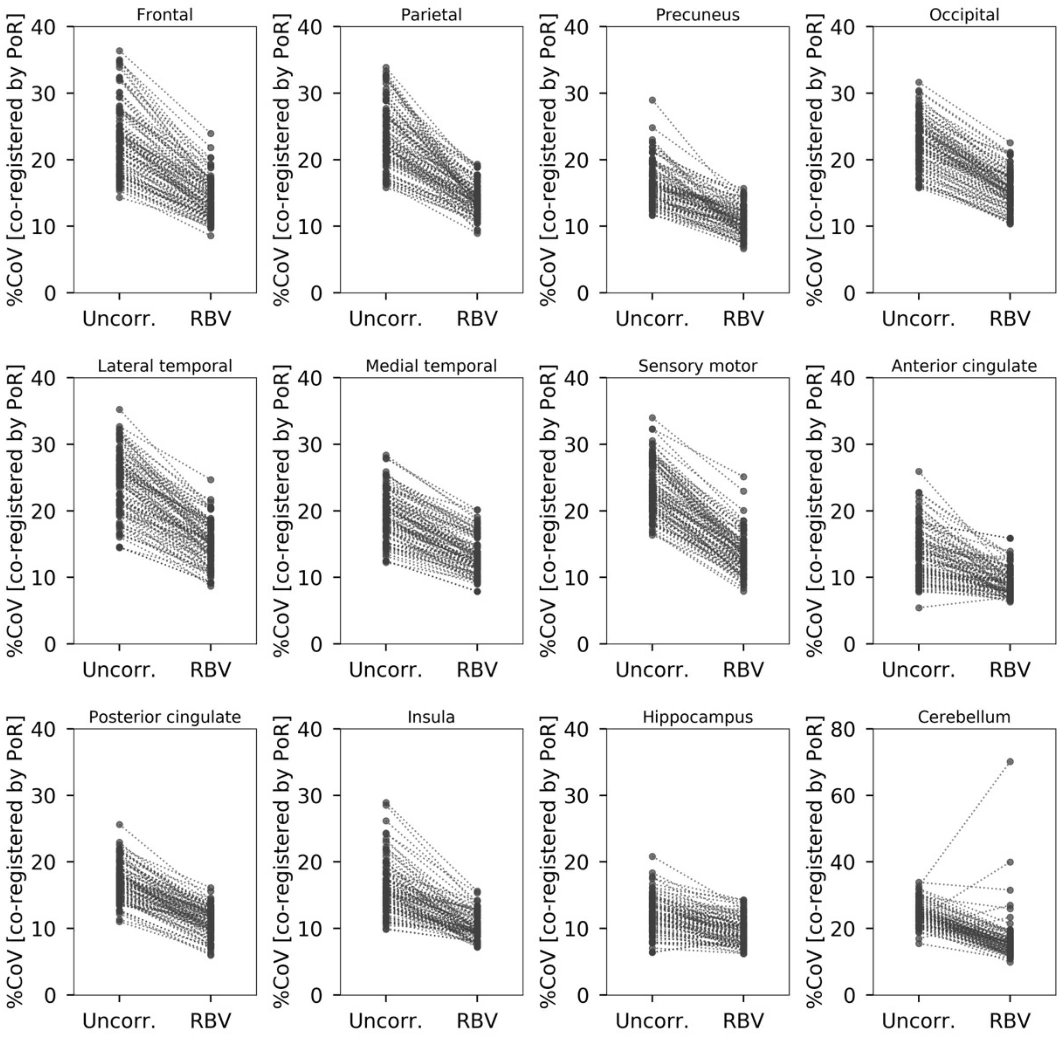


CoV, coefficient of variation; PoR, PVC-optimized registration; PV, partial volume; RBV, region-based voxelwise; SUV, standardized uptake value; VOI, volume of interest.

Figure S7. **% Differences in PV-corrected SUV between conventional registration and PoR for all VOIs.**

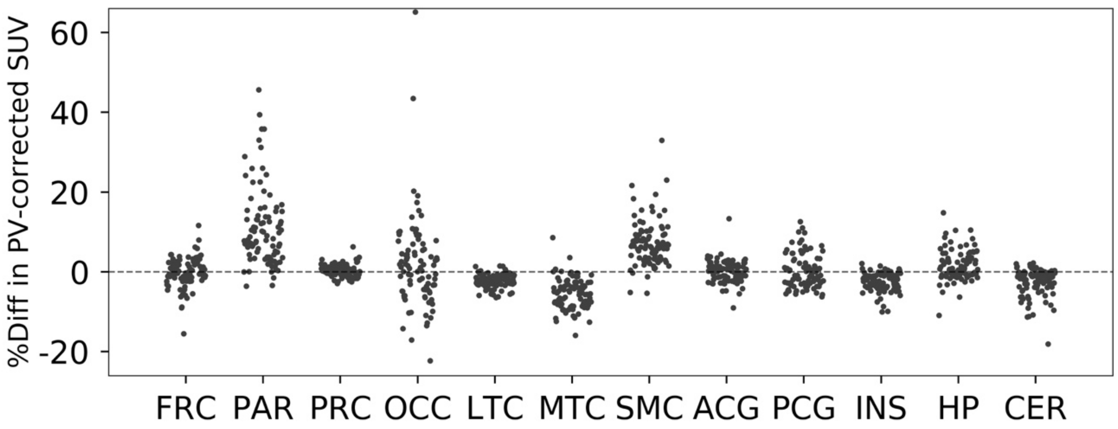


ACG, anterior cingulate cortex; CER, cerebellum; FRC, frontal cortex; HP, hippocampus; INS, insula; LTC, lateral temporal cortex; MTC, medial temporal cortex; OCC, occipital cortex; PAR, parietal cortex; PCG, posterior cingulate cortex; PoR, PVC-optimized registration; PRC, precuneus; PV, partial volume; RBV, region-based voxelwise method; SMC, sensory motor cortex; SUV, standardized uptake value; VOI, volume of interest.

Figure S8. **% Differences in PV-corrected SUVR between conventional registration and PoR for all VOIs.**

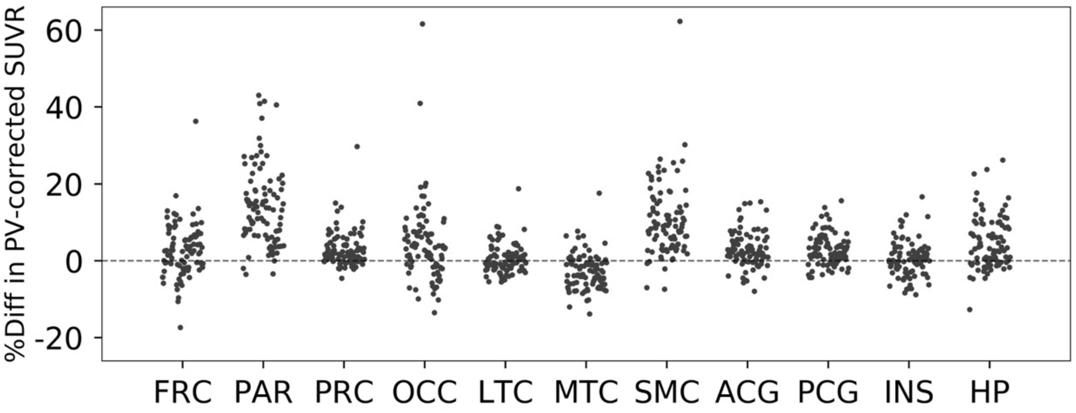


ACG, anterior cingulate cortex; CER, cerebellum; FRC, frontal cortex; HP, hippocampus; INS, insula; LTC, lateral temporal cortex; MTC, medial temporal cortex; OCC, occipital cortex; PAR, parietal cortex; PCG, posterior cingulate cortex; PoR, PVC-optimized registration; PRC, precuneus; PV, partial volume; RBV, region-based voxelwise method; SMC, sensory motor cortex; SUVR, standardized uptake value ratio; VOI, volume of interest.

Table S4. **Values in CoV on PV-uncorrected and PV-corrected SUV maps coregistered by conventional registration and PoR**

| VOI | PV-uncorrected | | PV-corrected | |
|---|---|---|---|---|
| | Conventional [median (Q1, Q3)] | PoR [median (Q1, Q3)] | Conventional [median (Q1, Q3)] | PoR [median (Q1, Q3)] |
| Frontal cortex | 23.1 (20.2, 25.8) | 22.9 (19.1, 27.0) | 13.6 (12.5, 15.8) | 13.5 (11.7, 16.1) |
| Parietal cortex | 25.7 (22.5, 29.8) | 22.9 (20.5, 26.7) | 15.7 (13.9, 17.4) | 13.6 (12.4, 15.3) |
| Precuneus | 16.0 (14.5, 18.6) | 16.0 (14.3, 18.1) | 10.6 (9.6, 11.9) | 10.6 (9.2, 12.2) |
| Occipital cortex | 23.8 (21.1, 27.2) | 22.9 (20.0, 25.2) | 17.3 (14.8, 19.4) | 15.1 (13.6, 17.2) |
| Lateral temporal cortex | 24.2 (20.5, 26.7) | 24.7 (20.9, 27.7) | 14.8 (13.1, 17.3) | 15.0 (12.9, 17.5) |
| Medial temporal cortex | 19.4 (16.7, 21.9) | 19.8 (17.2, 22.0) | 13.2 (11.4, 16.1) | 12.9 (11.2, 15.2) |
| Sensory motor cortex | 24.6 (22.4, 27.8) | 23.1 (20.4, 27.0) | 14.4 (12.5, 16.2) | 13.2 (11.4, 14.9) |
| Anterior cingulate cortex | 13.1 (10.6, 16.4) | 13.2 (10.5, 16.0) | 9.1 (7.7, 10.6) | 8.9 (7.8, 10.4) |
| Posterior cingulate cortex | 16.1 (14.7, 18.2) | 17.0 (15.2, 18.7) | 10.4 (9.0, 11.8) | 11.2 (9.7, 12.5) |
| Insula | 15.4 (13.1, 17.7) | 15.4 (13.4, 18.2) | 9.9 (8.9, 11.5) | 9.6 (8.9, 11.5) |
| Hippocampus | 12.3 (10.6, 14.3) | 11.8 (10.3, 13.6) | 10.5 (8.9, 12.3) | 9.4 (8.1, 10.7) |
| Cerebellum | 24.3 (22.6, 26.2) | 24.0 (22.2, 26.1) | 16.5 (14.1, 18.0) | 15.0 (13.1, 16.9) |

Q1 and Q3 refer to the 1$^{st}$ and 3$^{rd}$ quantiles of %difference, respectively. CoV, coefficient of variation; PoR, PVC-optimized registration; PV, partial volume; PVC, partial volume correction; SUV, standardized uptake value; VOI, volume of interest.

Table S5. **Values and their differences in intra-region CoV between PV-uncorrected and PV-corrected SUV maps, coregistered with PoR**

| VOI | PV-uncorrected [median (Q1, Q3)] | PV-corrected [median (Q1, Q3)] | ΔCoV [median (Q1, Q3)] | *p* value | Effect size |
|---|---|---|---|---|---|
| Frontal cortex | 22.9 (19.1, 27.0) | 13.5 (11.7, 16.1) | −8.4 (−12.0, −6.8) | < 0.001 | −2.73 |
| Parietal cortex | 22.9 (20.5, 26.7) | 13.6 (12.4, 15.3) | −8.5 (−12.7, −6.7) | < 0.001 | −2.32 |
| Precuneus | 16.0 (14.3, 18.1) | 10.6 (9.2, 12.2) | −5.1 (−7.2, −3.8) | < 0.001 | −1.87 |
| Occipital cortex | 22.9 (20.0, 25.2) | 15.1 (13.6, 17.2) | −7.2 (−9.0, −5.9) | < 0.001 | −3.41 |
| Lateral temporal cortex | 24.7 (20.9, 27.7) | 15.0 (12.9, 17.5) | −8.6 (−11.4, −7.1) | < 0.001 | −2.87 |
| Medial temporal cortex | 19.8 (17.2, 22.0) | 12.9 (11.2, 15.2) | −6.1 (−8.1, −4.8) | < 0.001 | −2.78 |
| Sensory motor cortex | 23.1 (20.4, 27.0) | 13.2 (11.4, 14.9) | −10.2 (−12.2, −8.3) | < 0.001 | −3.78 |
| Anterior cingulate cortex | 13.2 (10.5, 16.0) | 8.9 (7.8, 10.4) | −3.2 (−6.8, −1.8) | < 0.001 | −1.25 |
| Posterior cingulate cortex | 17.0 (15.2, 18.7) | 11.2 (9.7, 12.5) | −5.7 (−7.3, −4.5) | < 0.001 | −2.73 |
| Insula | 15.4 (13.4, 18.2) | 9.6 (8.9, 11.5) | −5.2 (−8.1, −3.4) | < 0.001 | −1.79 |
| Hippocampus | 11.8 (10.3, 13.6) | 9.4 (8.1, 10.7) | −2.3 (−3.5, −1.2) | < 0.001 | −1.25 |
| Cerebellum | 24.0 (22.2, 26.1) | 15.0 (13.1, 16.9) | −9.6 (−11.1, −7.2) | < 0.001 | −1.24 |

Q1 and Q3 refer to the 1st and 3rd quantiles of % difference, respectively. CoV, coefficient of variation; PoR, PVC-optimized registration; PV, partial volume; PVC, partial volume correction; SUV, standardized uptake value; VOI, volume of interest.

Table S6. **Differences in PV-corrected SUVs and the results of statistical analyses comparing conventional registration to PoR**

| VOI | Conventional [median (Q1, Q3)] | PoR [median (Q1, Q3)] | %difference [median (Q1, Q3)] | Effect size |
|---|---|---|---|---|
| Frontal cortex | 2.55 (1.16, 4.94) | 2.55 (1.14, 5.03) | 0.32 (−1.73, 2.40) | 0.35 |
| Parietal cortex | 2.25 (0.94, 4.64) | 2.48 (1.03, 4.95) | 8.56 (3.73, 14.36) | 0.83 |
| Precuneus | 3.07 (1.30, 6.37) | 3.11 (1.33, 6.36) | 0.17 (−0.60, 1.00) | 0.15 |
| Occipital cortex | 1.39 (0.75, 2.80) | 1.36 (0.74, 2.81) | 0.77 (−4.85, 5.47) | −0.21 |
| Lateral temporal cortex | 2.13 (1.07, 4.00) | 2.11 (1.05, 3.91) | −2.18 (−3.11, −1.17) | −0.78 |
| Medial temporal cortex | 2.30 (1.22, 3.61) | 2.18 (1.22, 3.57) | −5.38 (−8.17, −2.64) | −0.90 |
| Sensory motor cortex | 1.87 (0.88, 3.74) | 1.96 (0.93, 3.91) | 6.46 (3.38, 9.37) | 0.78 |
| Anterior cingulate cortex | 3.27 (1.50, 6.12) | 3.31 (1.51, 6.00) | 0.48 (−1.34, 1.38) | 0.00 |
| Posterior cingulate cortex | 3.21 (1.41, 5.97) | 3.16 (1.44, 5.92) | −1.00 (−2.81, 2.36) | −0.43 |
| Insula | 2.33 (1.31, 3.96) | 2.32 (1.28, 3.92) | −1.87 (−3.63, −0.87) | −0.81 |
| Hippocampus | 1.90 (1.01, 2.79) | 1.91 (1.01, 2.90) | 0.90 (−0.59, 3.58) | 0.51 |
| Cerebellum | 1.30 (0.75, 2.05) | 1.25 (0.74, 2.02) | −1.50 (−3.94, −0.23) | −0.62 |

Q1 and Q3 refer to the 1$^{st}$ and 3$^{rd}$ quantiles of %difference, respectively. PVC, partial volume correction; SUV, standardized uptake value

Table S7. **Differences in PV-corrected SUVRs and the results of statistical analyses comparing conventional registration to PoR**

| VOI | Conventional [median (Q1, Q3)] | PoR [median (Q1, Q3)] | %difference [median (Q1, Q3)] | Effect size |
|---|---|---|---|---|
| Frontal cortex | 2.48 (0.98, 3.18) | 2.57 (0.97, 3.32) | 2.67 (−1.33, 6.63) | 0.56 |
| Parietal cortex | 2.09 (0.79, 2.92) | 2.24 (0.90, 3.42) | 13.34 (6.60, 18.63) | 1.14 |
| Precuneus | 3.14 (1.19, 4.23) | 3.20 (1.18, 4.44) | 2.08 (0.27, 4.77) | 0.67 |
| Occipital cortex | 1.17 (0.68, 1.66) | 1.18 (0.73, 1.70) | 3.42 (−1.43, 7.52) | 0.47 |
| Lateral temporal cortex | 1.74 (1.00, 2.62) | 1.72 (0.98, 2.68) | −0.01 (−1.98, 1.88) | 0.33 |
| Medial temporal cortex | 1.75 (1.23, 2.37) | 1.64 (1.15, 2.36) | −3.55 (−6.01, −0.50) | −0.41 |
| Sensory motor cortex | 1.61 (0.85, 2.31) | 1.73 (0.91, 2.57) | 9.02 (4.08, 14.12) | 0.91 |
| Anterior cingulate cortex | 2.86 (1.49, 3.52) | 3.05 (1.49, 3.72) | 2.44 (0.52, 5.35) | 0.74 |
| Posterior cingulate cortex | 2.88 (1.49, 3.52) | 2.93 (1.52, 3.54) | 2.06 (0.45, 4.97) | 0.57 |
| Insula | 1.86 (1.26, 2.44) | 1.89 (1.23, 2.52) | 0.10 (−2.28, 2.49) | 0.30 |
| Hippocampus | 1.37 (1.30, 1.53) | 1.42 (1.33, 1.61) | 3.15 (−0.38, 9.32) | 0.67 |

Q1 and Q3 refer to the 1st and 3rd quantiles of %difference, respectively. PVC, partial volume correction; SUVR, standardized uptake value ratio (reference region: cerebellum)
